# Genetic diversity of Coxsackievirus A21 associated with sporadic cases of acute respiratory infections in Malaysia

**DOI:** 10.1186/s12879-021-06148-x

**Published:** 2021-05-17

**Authors:** Nur Izzati Supian, Kim Tien Ng, Jack Bee Chook, Yutaka Takebe, Kok Gan Chan, Kok Keng Tee

**Affiliations:** 1 Department of Medical Microbiology, Faculty of Medicine, University of Malaya, Kuala Lumpur, Malaysia; 2 Department of Medicine, Faculty of Medicine, University of Malaya, Kuala Lumpur, Malaysia; 3 Infectious Disease Translational Research Programme, Yong Loo Lin School of Medicine, National University of Singapore, Singapore, Singapore; 4 Department of Microbiology and Immunology, Yong Loo Lin School of Medicine, National University Health System, National University of Singapore, Singapore, Singapore; 5 Department of Medical Sciences, School of Healthcare and Medical Sciences, Sunway University, Bandar Sunway, Selangor Darul Ehsan Malaysia; 6 AIDS Research Center, National Institute of Infectious Diseases, Toyama, Shinjuku-ku, Tokyo, Japan; 7 Division of Genetics and Molecular Biology, Institute of Biological Sciences, Faculty of Science, University of Malaya, Kuala Lumpur, Malaysia; 8 International Genome Centre, Jiangsu University, Zhenjiang, China

**Keywords:** Enterovirus, Coxsackievirus, Acute respiratory tract infections, Outbreak

## Abstract

**Background:**

Coxsackievirus A21 (CVA21), a member of *Enterovirus C* from the *Picornaviridae* family, has been associated with respiratory illnesses in humans.

**Methods:**

A molecular epidemiological investigation of CVA21 was conducted among patients presenting with acute upper respiratory illnesses in the ambulatory settings between 2012 and 2014 in Kuala Lumpur, Malaysia.

**Results:**

Epidemiological surveillance of acute respiratory infections (*n* = 3935) showed low-level detection of CVA21 (0.08%, 1.4 cases/year) in Kuala Lumpur, with no clear seasonal distribution. Phylogenetic analysis of the new complete genomes showed close relationship with CVA21 strains from China and the United States. Spatio-temporal mapping of the *VP1* gene determined 2 major clusters circulating worldwide, with inter-country lineage migration and strain replacement occurring over time.

**Conclusions:**

The study highlights the emerging role of CVA21 in causing sporadic acute respiratory outbreaks.

**Supplementary Information:**

The online version contains supplementary material available at 10.1186/s12879-021-06148-x.

## Background

Enteroviruses (EV) are a diverse group of non-enveloped, positive-sense RNA virus which belongs to the family *Picornaviridae*. EV are subdivided into 15 species within the genus *Enterovirus*, which consists of *Enterovirus A* to *Enterovirus L* and *Rhinovirus A* to *Rhinovirus C* (RV-A to RV-C) [1, 2]. Coxsackievirus A21 (CVA21), which belongs to the *Enterovirus C* species, was first identified in a poliomyelitis outbreak in the summer of 1947 [3]. Similar to other enteroviruses, CVA21 have been associated with various respiratory, gastrointestinal and neurological symptoms in humans. In recent years, the role of CVA21 in causing sporadic outbreaks of respiratory infections in humans has become increasingly important [4, 5]. Most notably, in what appeared as the first reported large CVA21 outbreak in China, CVA21 was detected in about 57% of patients in a cluster of unexplained respiratory illnesses in 2016, with evidence of efficient human-to-human transmission [6]. Another recent outbreak reported that CVA21 was detected in 16% of military recruits with respiratory diseases, including febrile illness and pneumonia in the United States in 2002 [7].

Despite the clinical implications of enterovirus infection, CVA21 remains a rare serotype [8, 9] where routine diagnosis is very often not readily available in the healthcare settings which results in the under-reporting of CVA21 cases. Similarly, epidemiological surveillance and genetic characterization of CVA21 remain limited especially in the developing countries where respiratory disease burden is high [10]. In this study, we determined the distribution and analysed the first complete genomes of CVA21 among patients presenting with acute respiratory symptoms in Kuala Lumpur, Malaysia located in the Southeast Asia region.

## Methods

From March 2012 to May 2014, respiratory samples in the form of nasopharyngeal swab were collected from 3935 consenting outpatients presenting with acute respiratory tract infections at the Primary Care Clinics, University of Malaya Medical Centre in Kuala Lumpur, Malaysia. Specimens were screened for viral pathogens using the xTAG Respiratory Viral Panel FAST Assay (Luminex Molecular, Toronto, Canada) followed by specific enterovirus detection by PCR amplification and phylogenetic analysis [11]. The complete genomes of CVA21 strains were amplified and sequenced using 18 pairs of newly designed primers (Supplementary Table 1). The overlapping fragments were assembled to obtain the complete genome. The genomes were aligned with other previously reported CVA21 sequences (*n* = 21) retrieved from the GenBank (accessed on 15 December 2020). Phylogenetic trees were constructed by the neighbour-joining method based on the Kimura two-parameter model implemented in MEGA X [12]. The reliability of the branching orders was analysed by bootstrap analysis of 1000 replicates. Similarity plot analysis was performed to investigate the possible occurrence of recombination in the genomes. Neighbour-joining tree was also reconstructed based on the *VP1* region (*n* = 54; accessed on 15 December 2020), using the approach employed in the complete genome analysis. This study was approved by the University Malaya Medical Centre Medical Ethics Committee (MEC890.1).

## Results and discussion

During the epidemiological surveillance, a total of 3/3935 (0.08%) cases of CVA21 infection were detected among outpatients presenting with acute respiratory symptoms (Table 1). Two CVA21 cases were identified in May 2012; patient 12MYKLU412 was a 52-year-old female presented with mild respiratory symptoms, and patient 12MYKLU434 was a 16-year-old male with severe headache and sore throat. Another patient 14MYKLU3370 was a 26-year-old female presented on January 2014 with severe headache and nasal congestion. No distinct distribution pattern or seasonality was observed, indicative of the sporadic nature of CVA21 infections [4, 8, 9]. However, the increased detection of CVA21 in May 2012 may be suggestive of a modest outbreak that occurred undetected in the population, although more data is needed to confirm it. Overall, the rate of detection was estimated at 1.4 cases/year in Kuala Lumpur, Malaysia, as compared to 0.65, 0.77, 1.2 and 9.2 cases/year reported in Japan, Singapore, United States and China, respectively [4, 5, 8, 13].

**Table 1:** Demographic and clinical symptoms of patients infected with CVA21

Patient ID	Collection date	Demographic profile		Upper respiratory symptoms presented
		Age	Sex	
12MYKLU412	2 May 2012	52	Female	Cough, sore throat, sneezing, nasal discharge
12MYKLU434	4 May 2012	16	Male	Severe headache and sore throat, cough, hoarseness of voice, sneezing, nasal congestion with discharge
14MYKLU3370	13 January 2014	26	Female	Severe headache and nasal congestion, cough, sore throat, hoarseness of voice, sneezing, nasal discharge, myalgia

**Fig. 1:**
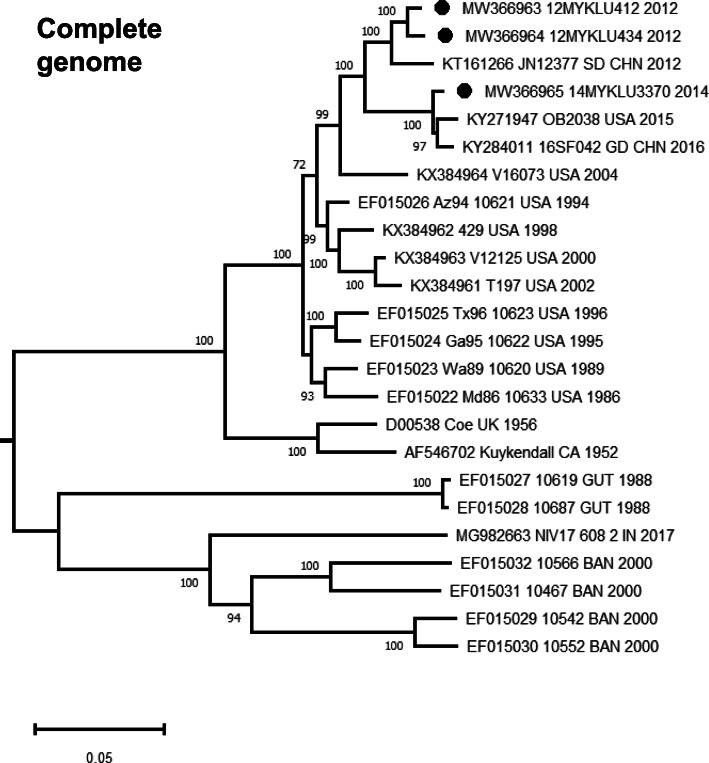
Phylogenetic analysis of CVA21 complete genomes. Phylogenetic tree was reconstructed by the neighbour-joining method based on all available complete genomes of CVA21 (*n* = 24) using MEGA X. The reliability of the branching nodes was assessed by bootstrap analysis of 1000 replicates. Bootstrap values (> 70%) are shown at the respective branches. Scale bar represents a genetic distance of 0.05 substitutions/site. Accession number, location and year of isolation are shown for previously published CVA21 genomes in GenBank. The newly generated sequences in this study are indicated in circle

The three complete CVA21 genomes were 7339 nucleotides (nt) in length, which consist of a 690-nt 5′ untranslated region (UTR), followed by a 6621-nt polyprotein region encoding an open reading frame of 2207 codons, and the 3′ UTR region. The genome organization of these genomes was similar to that of previously reported CVA21 prototype strains [14]. The average percentage of bases for all three genomes were 30.4% A, 22.3% G, 22.1% C and 25.2% U. Phylogenetic analysis based on all available CVA21 complete genomes in GenBank showed that strains from 12MYKLU412 and 12MYKLU434 were grouped within a cluster (1.07% mean genetic distance) with the JN12377/SD/CHN/2012 strain (accession no. KT161266) isolated from environmental sewage in Shandong Province, China in 2012 (Fig. 1). Strain isolated from 14MYKLU3370 meanwhile was closely related to a recent outbreak strain 16SF042/CHN/2016 (accession no. KY284011) isolated in Guangdong Province, China and the USA/TN/2015-OB2038 strain (accession no. KY271947) isolated from stool in Tennessee, United States. No significant evidence of recombination was detected in these genomes in the similarity plot analysis (data not shown).

**Fig. 2:**
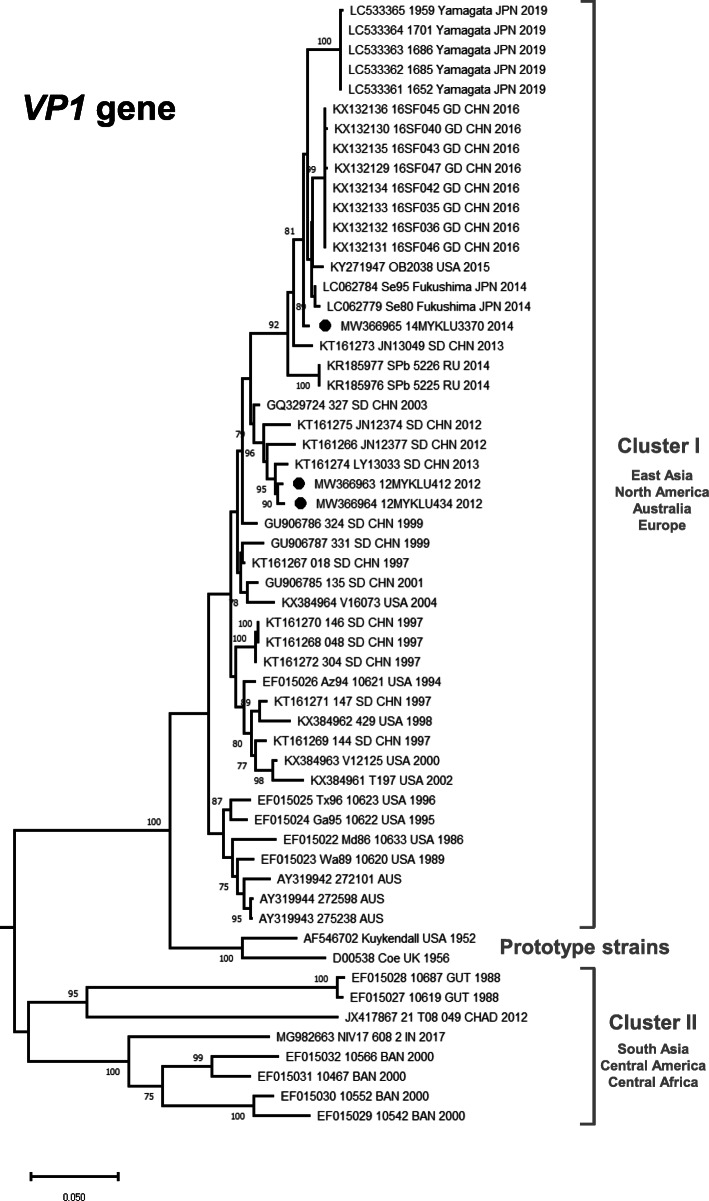
Phylogenetic analysis of the global CVA21 *VP1* gene. Phylogenetic tree was reconstructed by the neighbour-joining method based on *n* = 54 available complete CVA21 *VP1* gene sequences (894 bp), equivalent to position 2497–3390 of the prototype strain (GenBank accession no. AF546702). CVA21 strains grouped within Cluster I (East Asia, North America, Australia and Europe) and Cluster II (South Asia, Central America and Central Africa) are indicated by brackets. The reliability of the branching nodes was assessed by bootstrap analysis of 1000 replicates. Bootstrap values (> 70%) are shown at the respective branches. Scale bar represents a genetic distance of 0.05 substitutions/site. GenBank accession number, location and year of isolation are shown for previously reported CVA21 strains. The newly generated sequences in this study are indicated in circle

In order to attain a greater spatio-temporal resolution for the global CVA21 strains, phylogenetic analysis based on all available *VP1* coding region (*n* = 54) showed that the contemporary CVA21 strains can be divided into two clusters; cluster I and II (Fig. 2) [9]. Cluster I comprised of CVA21 strains isolated from East Asia (China, Japan and Malaysia), North America (United States), Australia and Europe (Russia). Of note, recent outbreak strains in Guangdong Province, China (2016) and Yamagata, Japan (2019) were characterized by profound founding effects with limited genetic diversity within the respective clusters. In addition, weak or limited spatial distribution is observed among CVA21 circulating worldwide with frequent strain mixing, suggesting active lineage mobility between regions. Interestingly, phylogenies within cluster I were characterized by a “ladder-like” structure resulting from the continual turnover of viral lineages through time. Cluster II meanwhile comprised of CVA21 strains originated from South Asia (India and Bangladesh), Central America (Guatemala) and Central Africa (Chad) (Fig. 2). Overall, the tree topology of the complete *VP1* region observed was similar to that of the complete genomes in Fig. 1.

It is important to note that the surveillance and genomic data presented in the study contain several inherent limitations, such as the relatively old sampling period, limited sampling site and the inability to establish the seasonality of CVA21 due to the low detection rate. Therefore, the recent activity of CVA21 in the region remains unclear, highlighting the need for continuous sentinel surveillance involving multiple sites and timely reporting in order to facilitate effective outbreak response strategies.

## Conclusions

In summary, we studied and characterised the distribution and complete genomes of CVA21 isolated from patients presenting with acute respiratory symptoms in Malaysia, highlighting the emerging role of CVA21 in causing low-level but recurrent respiratory outbreaks. The evolutionary dynamics of global CVA21 strains showed widespread distribution of at least two major phylogenetic clusters worldwide.

## Supplementary Information

**Additional file 1: Supplementary Table 1.** Primer sequences used for complete genome amplification of Coxsackievirus A21.

## Data Availability

The CVA21 genome sequences are available in GenBank under the following accession numbers: MW366963-MW366965. The sample collection dates and isolation source are available in the National Center for Biotechnology Information (NCBI) public database. Public access to the database is open.

## Abbreviations

CVA21: Coxsackievirus A21
EV: Enteroviruses
nt: Nucleotides
PCR: Polymerase chain reaction
UTR: Untranslated region
