# Supplementary material for: Genetic diversity of Coxsackievirus A21 associated with sporadic cases of acute respiratory infections in Malaysia
Source: BMC Infect Dis. 2021 May 17;21:446. doi: 10.1186/s12879-021-06148-x (PMC8130276; doi:10.1186/s12879-021-06148-x)
Supplement: Supplementary file 1 — Additional file 1: Supplementary Table 1. Primer sequences used for complete genome amplification of Coxsackievirus A21. [file 12879_2021_6148_MOESM1_ESM.docx]

Supplementary Table 1. Primer sequences used for complete genome amplification of Coxsackievirus A21

| Fragment | PCR cycle | Primer | Primer sequence  (5' to 3') | Nucleotide position |
| --- | --- | --- | --- | --- |
| 5'UTR | 1^st^ | Forward 5' | TTAAAACAGCYYKKGGGTTGYTCC | 1 to 24 |
|  |  | Reverse 5'1 | AYCCACAYGCTTCCACRTTDGGC | 946 to 924 |
|  | 2^nd^ | Forward 5' | TTAAAACAGCYYKKGGGTTGYTCC | 1 to 24 |
|  |  | Reverse 5'2 | TTGTCTRGTRGCSGARTTACTYGC | 846 to 823 |
| 1 | 1^st^ | Frag1 F1 | TATTGTYGCTTAYGGTGARTGGC | 1014 to 1036 |
|  |  | Frag1 R1 | AGAAAGGTGAACCTRATGGACCC | 2090 to 2068 |
|  | 2^nd^ | Frag1 F2 | TCAGARGCWAAYCCRGTAGATGC | 1054 to 1076 |
|  |  | Frag1 R2 | TYARTCGTTTRTCAGAAGCAGGTG | 2020 to 1997 |
| 2 | 1^st^ | Frag2 F1 | ATGGAACTRGCYGARATTG | 1867 to 1885 |
|  |  | Frag2 R1 | CCRTCRTARAAGTGTGAATAGGC | 3075 to 3053 |
|  | 2^nd^ | Frag2 F2 | AATGAAYGCAGTGGACGGG | 1899 to 1917 |
|  |  | Frag2 R2 | TAAGGAATTGACATCCGTGG | 3036 to 3017 |
| 3 | 1^st^ | Frag3 F1 | AGTACAGCYAGTGGRGAAGTGCG | 2873 to 2895 |
|  |  | Frag3 R1 | AYTCCACCACCATTAAATCCC | 3482 to 3462 |
|  | 2^nd^ | Frag3 F2 | ATGTAYATTCCACCAGGGGC | 2915 to 2934 |
|  |  | Frag3 R2 | TACACTGCYTTGTTYTGRTGRCC | 3381 to 3359 |
| 4 | 1^st^ | Frag4 F1 | TTTGGAGTYYTAGCRGTYAGAGC | 3152 50 3174 |
|  |  | Frag4 R1 | TCARCTTGGTGAGAAATTCCACC | 4241 to 4219 |
|  | 2^nd^ | Frag4 F2 | AAAACACATTCGGTGYTGGTGCC | 3232 to 3254 |
|  |  | Frag4 R2 | TTTYTTCATCCACCCATCACC | 4111 to 4091 |

| 5 | 1^st^ | Frag5 F1 | TGCAACACTAGCAYTRCTYGGG | 3994 to 4015 |
| --- | --- | --- | --- | --- |
|  |  | Frag5 R1 | TCYACRGTGAAYCTYTGTCTGG | 5001 to 4980 |
|  | 2^nd^ | Frag5 F2 | ACTAGAGATTCCTYAYGTGMTGC | 4063 to 4085 |
|  |  | Frag5 R2 | TTWCCACARACRAGAGGGC | 4953 to 4935 |
|  | 1^st^ | Frag6 F1 | TCAYAGTGATGCCTTAACCAGACG | 4789 to 4812 |
| 6 |  | Frag6 R1 | ACCTCATCCAYTGRATTTCGCC | 5975 to 5954 |
|  | 2^nd^ | Frag6 F2 | TGTCWGAACAYTCARTCAAAGGC | 4842 to 4864 |
|  |  | Frag6 R2 | AGTRCAAGTRATRATTCCYCCGC | 5866 to 5844 |
|  | 1^st^ | Frag7 F1 | AACYAAYGATGGAGTGTTGATCG | 5692 to 5714 |
| 7 |  | Frag7 R1 | TAGTCTTTTCCTGATTGGGCTAGG | 7002 to 6979 |
|  | 2^nd^ | Frag7 F2 | AGGGRTATCTYAATCTCAGTGGACG | 5769 to 5793 |
|  |  | Frag7 R2 | AGCTATCACRTCATCACCATAGGC | 6949 to 6926 |
|  | 1^st^ | Frag8 F1 | TGGAGYAAGATACCAGTRYTAATGG | 6605 to 6629 |
| 8 |  | Frag8 R | TTTACCCCTACAACAGTATAACCC | 7390 to 7367 |
|  | 2^nd^ | Frag8 F2 | ATGCCATCYGGCTGYTCTGGC | 6809 to 6829 |
|  |  | Frag8 R | TTTACCCCTACAACAGTATAACCC | 7390 to 7367 |

R: A or G, Y: C or T, M: A or C, K: G or T, S: C or G, W: A or T, D: A or G or T
